# Preliminary Investigation of a Mobile Nutrition Literacy Website for Parents and Young Children

**DOI:** 10.3389/fnut.2018.00129

**Published:** 2018-12-18

**Authors:** Heather D. Gibbs, Juliana Camargo, Susana Patton, Jamie Zoellner, Yvonnes Chen, Ana Paula Cupertino, Susan Harvey, Byron Gajewski, Debra K. Sullivan

**Affiliations:** ^1^Department of Dietetics & Nutrition, University of Kansas Medical Center, Kansas City, KS, United States; ^2^Department of Pediatrics, University of Kansas Medical Center, Kansas City, KS, United States; ^3^Department of Public Health Science, University of Virginia, Charlottesville, VA, United States; ^4^School of Journalism and Mass Communications, University of Kansas, Lawrence, KS, United States; ^5^John Theurer Cancer Center, Hackensack Meridian Health, Hackensack, NJ, United States; ^6^Department of Health, Sport, and Exercise Sciences, University of Kansas, Lawrence, KS, United States; ^7^Department of Biostatistics, University of Kansas Medical Center, Kansas City, KS, United States

**Keywords:** health communication, literacy, nutrition, patient education, parenting

## Abstract

Parental nutrition literacy (PNL) correlates positively with child diet quality, but interventions for improving PNL are lacking. “Nutricity” is a novel bilingual (English/Spanish) mobile tool designed by the research team to engage parents and young children to interact with nutrition information to make nutrition decisions. The purpose of this study was to inform a future intervention through (1) assessing parental likability of Nutricity, and (2) collecting perceptions of pediatric clinic personnel on the feasibility of introducing Nutricity in pediatric clinics. PNL scores and feedback about Nutricity were collected using mixed methods from 15 English-speaking and 15 Spanish-speaking parents of 1–5 year-old children. Three parents from each language group provided additional feedback via semi-structured interviews. Interviews with 11 pediatric clinic personnel were also conducted to anticipate barriers and formulate strategies for implementing Nutricity as a clinic-based intervention. Nutricity was liked by both language groups and across all PNL levels, with a mean rating of 4.6 on a 5-point scale. Clinic personnel interviews affirmed need for and feasibility of offering Nutricity in clinics.

## Introduction

Diets of US children fall short of recommendations, resulting in excess consumption of some nutrients and inadequate intake of others (1). Dietary patterns established in infancy persist long-term, making early childhood a critical time to set the trajectory toward a healthy diet (2).

Parents have tremendous influence upon their child's diet. They determine food availability and accessibility, model eating behavior, exert feeding styles influencing child eating behavior, as well as determine context for family meals, frequency of dining out, and portion sizes served and/or eaten (3). Yet children also influence parental feeding behavior. For example, child fear and food rejection when presented with a new vegetable may impair repeated exposure by parents (4). Because children and parents work bi-directionally to form eating habits, interventions to improve diet quality in young children should include both parents and children.

Maternal nutrition knowledge is an important mediator of interventions to improve child diet quality (3). Nutrition literacy is a functional type of knowledge, requiring an ability to use nutrition information and skills to make nutrition decisions. Our study of parent-child dyads found a strong positive relationship between parental nutrition literacy and child diet quality (5). These findings suggest improving parent nutrition literacy may improve the diet quality of children.

Primary health care settings are a preferred avenue for parents to receive health-related information (6). However, between waiting room and exam room, many patients wait 30 min or more before meeting with their primary care provider, and long waits decrease patient satisfaction and reduce motivation for seeking health services (7). While recommended well-care visits are frequent for young children, primary care providers have time constraints limiting preventive education including nutrition.

To address this gap in nutrition care, the research team designed a mobile website to improve parental nutrition literacy intended to be introduced to parents and young children within pediatric clinics. In this formative study to inform a future intervention, our purposes were: (1) Assess the likability, usability, and engagement of parents and children with a mobile bilingual (English/Spanish) website created by the research team entitled, “Nutricity;” and (2) Assess the feasibility of introducing *Nutricity* in a pediatric waiting room setting. Presented as a cartoon map of a town, the title of *Nutricity* was formed from the words “nutrition” and “city,” a term that could apply for both English and Spanish users, while also sounding like “electricity,” as in the energy-delivering function of nutrition.

## Materials and Methods

### Study Design

We conducted a prospective, mixed methods study to evaluate Nutricity, designed for improving nutrition literacy of parents and young children between ages 1–5 years. The Institutional Review Board at the University of Kansas Medical Center approved the research protocol. All procedures were in accordance with ethical standards described in the Declaration of Helsinki.

### Nutricity

*Nutricity* uses a combination of communication techniques consistent with health literacy intervention research, (e.g., teach-back and chunk-and-check) (8). **Five** components of nutrition literacy are addressed, including ability to comprehend nutrition text, portioning foods appropriately, using food labels to make nutrition decisions, grouping foods into like categories, and choosing between similar foods (5). Content organization was inspired by the conversation map design, allowing users to choose the environmental context for interacting with food and nutrition information (9). Environments include the grocery store, home, and restaurants which are frequent locations where nutrition decisions are made for/with children. The goal of its design was to engage users to interact with the nutrition content, and thereby build capacity with nutrition information. Each location includes a 3-pronged approach (Supplementary Material): (1) Short videos forming a nutrition base, (2) Content-based games for kids and parents to play together, and (3) Interactive quizzes for parent practice of nutrition skills.

### Data Collection Procedures

Parents were recruited by clinic personnel referral from **two** pediatric clinics to evaluate *Nutricity*. Eligibility required participants to speak/read in English/Spanish, be parent or caregiver between 18 and 64 years with a child between 1 and 5 years, and identify as the primary food decision-maker for the home. Those with overt cognitive/psychiatric illnesses, visual impairments precluding reading from a tablet, or with children requiring highly restrictive diets (e.g., type 1 diabetes) were excluded.

Bilingual research assistants conducted study visits and data verification. After consent, participants completed a demographic survey and validated 42-item Nutrition Literacy Assessment Instrument (NLit) in English (10) or Spanish (11). NLit scores were interpreted as ≤28 = “likelihood of poor nutrition literacy,” 29–38 = “possibility of poor nutrition literacy,” and ≥39 = “likelihood of good nutrition literacy.” The NLit was chosen over other general health literacy tools due to its specificity to nutrition literacy, the target construct of *Nutricity*. Next, participants viewed the website on a study tablet for 30 min, preferably with their children, and then completed a 20-item survey adapted from Silk et al. (12) to evaluate *Nutricity's* likability. The survey consisted of 14 Likert-scale and **six** open-ended questions. A subsample (*n* = 6) completed semi-structured interviews to assess ease of use, engagement, and barriers to using *Nutricity*. Additionally, aggregated website usage was collected on video and quiz portions of *Nutricity* (not games due to technical difficulties) to identify most and least viewed sections.

Pediatricians and staff were interviewed to determine interest, barriers and strategies for integrating *Nutricity* into the pediatric clinic setting. A sampling frame of 3 interviews per topic to be completed, resulted in a sample of 9 interviews. The research team designed a 7-item semi-structured interview guide. Interviews were digitally recorded, transcribed, and reviewed for accuracy prior to analysis.

### Data Analysis

Surveys and website reports were evaluated with descriptive statistics and differences (*t*-tests) or associations (Chi-square/Fisher's Exact and Pearson's correlation) among demographic groups (education, income, and language) and nutrition literacy levels using SPSS (release 22.0, IBM Corp., Armonk, NY, 2013). Transcripts and field notes from all interviews were analyzed using the constant comparative method (13) and data triangulation (14) to identify recurring themes. Interviews conducted in Spanish were transcribed into Spanish and then translated to English by trained bilingual research assistants. Translation to English provided comparison of the interviews with English cohort interviews. Still, bilingual research assistants participated in the analysis of the interviews in English, to avoid any misinterpretation due to cultural differences. To limit bias, **two** researchers coded transcripts separately and met with the **first** author to discuss major themes and reach saturation of the themes.

## Results

### Findings From Parents

Thirty parents participated between two language groups, English (*n* = 15) and Spanish (*n* = 15). Sample characteristics and their likability ratings of *Nutricity* are shown in Table 1. Most were Hispanic (*n* = 19, 63%), female (*n* = 29, 97%), and participated in income assistance programs (*n* = 18, 60%). Participants had a “possibility of poor nutrition literacy” (μ = 31.3) and differed between groups with English speakers scoring significantly higher while mean scores of Spanish-speakers were considered “likelihood of poor nutrition literacy” (34.6 and 28.1, English and Spanish, respectively; *p* = 0.009). Positive relationship was seen between nutrition literacy and education (*r* = 0.498, *p* = 0.005) and nutrition literacy and income (*r* = 0.529, *p* = 0.003).

**Table 1:** Characteristics of parent participants and their likability ratings of *Nutricity*, a mobile nutrition literacy intervention platform.

**Characteristic**	**English cohort (*n* = 15)**	**Spanish cohort (*n* = 15)**	**Overall (*n* = 30)**	**Comparisons *p*-value and/or *X*^**2**^**
Ethnicity/Race, *n* (% of cohort)				
Hispanic White	2 (13%)	5 (33%)	7 (23%)	N/A
Hispanic Other	2 (13%)	10 (57%)	12 (40%)	
Non-Hispanic White	8 (53%)	0	8 (27%)	
Non-Hispanic Black	3 (20%)	0	3 (10%)	
Age, μ years (SD); *t*-test	29.3 (9.7)	31.8 (7.1)	30.6 (8.4)	*p* = 0.43
Education, *n* (% of cohort); *X*^2^				*X^2^*(2, *N* = 30) = 5.89, *p* = 0.053
<high school	2 (13%)	7 (47%)	9 (30%)	
High school/GED	4 (27%)	5 (33%)	9 (30%)	
Some college and higher	9 (60%)	3 (20%)	12 (40%)	
Income^a^, *n* (% of cohort); *X*^2^				*X^2^* (2, *N* = 30) = 8.09, *p* = 0.018^*^
<$25,000	4 (27%)	11 (73%)	15 (50%)	
$25,000–74,999	7 (47%)	4 (27%)	11 (37%)	
>$75,000	4 (27%)	0	4 (13%)	
Nutrition Literacy^b^, μ score (SD); *t*-test	34.6 (7.0)	28.1 (5.5)	31.3 (7.0)	*p* = 0.009^**^
Nutrition Literacy Category, *n* (%), *X^2^*				*X*^2^ (2, *N* = 30) = 10.68, *p* = 0.005^**^
1. “Likelihood of poor nutrition literacy,” (≤28 points)	2 (13%)	8 (53%)	10 (33%)	
2. “Possibility of poor nutrition literacy,” *n* (%) (29–38 points)	6 (40%)	7 (47%)	13 (43%)	
3. “Likelihood of good nutrition literacy,” *n* (%) (≥39 points)	7 (47%)	0	7 (23%)	
Overall Likability rating^c^, μ (SD); t-test	4.6 (0.3)	4.5 (0.3)	4.6 (0.3)	*p* = 0.45
Liking	4.7 (0.2)	4.1 (0.3)	4.4 (0.3)	*p* < 0.001^**^
Attention	4.7 (0.4)	4.9 (0.2)	4.8 (0.2)	*p* = 0.08
Intention	4.4 (0.6)	4.7 (0.3)	4.5 (0.3)	*p* = 0.06
Understanding	4.8 (0.3)	4.5 (0.4)	4.7 (0.4)	*p* = 0.60

* p < 0.05;

** p < 0.01

a *Median income by zipcode was used as a proxy for annual household income for participants who did not report annual incomes (n = 6)*.

b *Nutrition Literacy measured using the Nutrition Literacy Assessment Instrument in English or Spanish; Maximum score = 42 points*.

c *Likability assessed by survey adapted from Silk et al. Data shown for 14, Likert-style items participants ranked on scale of “1” (strongly disagree) to “5” (strongly agree)*.

Overall likability of *Nutricity* was rated 4.6 on a 5-point scale (1 = strongly disagree, 5 = strongly agree), and did not differ between groups (4.6 and 4.5, English and Spanish, respectively; *p* = 0.45). While significant difference was seen between quantitative ratings for “Liking,” differences in liking were not apparent in the qualitative interviews. No relationship was seen between nutrition literacy and overall likability (*r* = −0.001, *p* = 0.997).

Analysis of answers to open-ended survey questions indicate children who attended with parents primarily watched videos or played games, and most indicated it was “easy” or “very easy” for children to participate (*n* = 17). However, others felt it was more difficult for children (*n* = 7), and suggestions were made to improve touch-screen navigation to activities, incorporate more animation, and add more child actors in videos.

Parents who completed semi-structured interviews (3 English, 3 Spanish) gave non-significantly lower likability ratings than the overall sample (m = 4.4, SD = 0.18, *p* = 0.23) and indicated strong support of *Nutricity* in the interviews. Information presented in *Nutricity* was considered easy to understand by most interviewed (*n* = 5).

One suggested “*extremely easy without sounding condescending*.” (English-speaking, likelihood of good nutrition literacy)

Another expressed some difficulty completing a quiz stating, “*To read the information of every product of how much calories and everything, to make the sums if I have to subtract or I have to add, all of this was difficult for me.”* (Spanish-speaking, likelihood of poor nutrition literacy)

All six indicated they would use *Nutricity* if given continued access. Regardless of nutrition literacy level, all reported new information learned. As examples:

One noted, “*I eat out a lot because they do not like to eat many things. Now I learned that you can change the fries for something else to make the plate better, right?”* (Spanish-speaking, likelihood of poor nutrition literacy)

Another said “*I can try [vegetables] every time and not force them to eat it. ‘Cause by me forcing them to eat…it's just going to make it worse. But if I eat the vegetable, maybe they'll learn how to eat it too.”* (English-speaking, possibility of poor nutrition literacy)

Child participation reported by parents occurred in 12 (80%) English group visits and averaged 20 min viewing time. For the Spanish group, 6 children (40%) viewed *Nutricity* averaging 21 min. Website usage reports indicate videos were most viewed, although participants in both groups viewed all sections.

### Findings From Key Stakeholder Interviews

Eleven clinic personnel, including pediatricians (*n* = 7), nurse managers (*n* = 3), and one nurse from four area metro clinics participated in interviews (Table 2). Most noted nutrition care occurred within growth chart discussions with parents and/or obesity prevention. Regarding feasibility of introducing Nutricity during exam wait time, most shared similar sentiments such as “They are already using smartphones and tablets, so they may as well be doing something worthwhile,” and some noted perhaps it would decrease the number of children who play with the examination equipment while waiting. Most were concerned they have time constraints during patient care limiting their recruitment of patients. However, some offered alternative suggestions, such as flyers, patient portals, and the electronic medical record.

**Table 2:** Content analysis of interviews (*n* = 9) with pediatricians and clinic staff from four metro clinics.

**Interview question**	**Themes uncovered by interviews**	**Potential action/implication**
What do you observe caregivers/children doing while they wait to see the physician?	Parents and/or children on smartphones (*n* = 6) Kids minimally supervised (*n* = 4)—playing with clinic equipment, climbing on furniture, etc.	Using smartphones and/or tablets for educational purposes capitalizes on technology already used by clinic clientele. If some caregivers provide minimal supervision while waiting for exams, a mobile website should be toddler-friendly enough to require minimal caregiver supervision.
Are there entertainment options and/or educational materials?	Books, wall puzzles/toys, magazines for entertainment (*n* = 9); “Fitastic,” “Healthy Hawks,” and Bright Futures educational handouts (*n* = 9)	Children and parents are often not using available entertainment/educational offerings while waiting for physicians.
How long do they typically wait in the waiting room?	Estimates ranged 0 – 25 minutes (*n* = 7)	Wait time in waiting rooms may not be long enough to offer an educational intervention.
How long do they typically wait in the exam room?	Estimates ranged 10 – 45 minutes (*n* = 9)	Wait time in exam rooms may be a better avenue for offering an educational intervention.
How important is nutrition education for parents of young children?	Part of general guidance/within growth chart conversation (*n* = 4); Need for general nutrition knowledge (*n* = 2); Important for obesity prevention and treatment (*n* = 2)	Importance of nutrition education is largely recognized in the context of growth/obesity.
If we can recruit caregivers/parents from your clinic for an intervention study as we've described, how would you prefer we approach potential participants?	Must be researcher initiated due to inadequate time of medical providers (*n* = 6) Technology: use electronic medical record to identify qualified patients (*n* = 3); flag charts using a “smartphrase” to alert qualified patients of study opportunity (*n* = 3); email qualified patients	Providers are supportive of clinic-based nutrition education interventions but cannot add recruitment to their workload.
How feasible would it be for caregivers/children to use the website (on a tablet) while waiting for an appointment?	Very feasible, if clinic flow isn't slowed (*n* = 6); Not in waiting room due to other children not participating also wanting iPads (*n* = 1)	
Are you interested in a potential partnership with the research team to offer the proposed intervention?	Yes (*n* = 9)	
What potential barriers exist for your clientele to participate in the proposed intervention?	Home internet access (*n* = 6), although smartphones are widely used by clientele (*n* = 3); Healthy food access (*n* = 2) No shows/non-compliance (*n* = 3); Technology literacy/health literacy (*n* = 4)	Mobile technology-based nutrition education interventions should be smartphone-friendly or provide internet and device access.
Do you have any suggestions for how to address these identified barriers?	Monetary incentives (*n* = 5) Anticipating no-shows: Do as much as possible at baseline appointment and oversample (*n* = 1) Food access issues: Provide SNAP info (*n* = 1) Technology literacy: Give QR code for easy access to Nutricity at home (*n* = 1); Demo tablet use (*n* = 1) Health literacy: Provide assistance for reading questionnaires and use teach-back (*n* = 1)	Participants must be incentivized; Researchers should anticipate widely ranging barriers and work to make participation convenient; The intervention should be designed to accommodate individuals with low health literacy.

## Discussion

Across all levels of nutrition literacy and both language groups, parents provided strong support, and likability of *Nutricity*. Likewise, pediatric clinic personnel affirmed the feasibility of incorporating *Nutricity* as a wait-time intervention within clinics. Importantly, NLit scores of the parent sample demonstrated nutrition literacy deficits, indicating successful recruitment of the target population.

While differences between NLit scores of language groups were observed, national assessment data indicate low health literacy is more common among Hispanics (15). Mediators of health literacy, including low socioeconomic and educational status, were also more often present in the Spanish group.

Clinic provider feedback affirmed *Nutricity* as an ideal vehicle for delivering preventive education otherwise not obtained by most patients. Exposure to educational material while waiting for an appointment could improve patient satisfaction while also prompting questions for the physician during the exam, potentially improving patient-provider communication (8). Health promotion apps are increasing in popularity due to availability and use of mobile technology, and interventions utilizing these technologies have improved dietary behaviors (16). Likewise, because *Nutricity* is designed for any internet-accessing device, learning could continue beyond the clinic. This includes smartphones, which a recent consumer survey indicates are owned by ~75–92% of the US population, depending upon age, race, and educational attainment (17). Although parents are crucial participants in developing healthy eating habits in their children, they are difficult to engage in nutrition interventions. Introduced as a wait-time intervention, *Nutricity* may overcome this barrier by reaching parents where they are common participants in their child's health care. Using developmentally appropriate games to engage young children may increase knowledge and improve behaviors, although further research in this area is needed (18).

Data is formative, involving a small, non-generalizable sample. While the sample's representativeness was not measured, its distribution demonstrated targeted demographics. Nutricity demonstrates promise as a bilingual mobile nutrition literacy intervention for parents of young children feasible to introduce in pediatric clinics. Further research should explore child perceptions of Nutricity, its effectiveness for improving nutrition literacy and implementation in primary health clinics.

## Data Availability Statement

The raw data supporting the conclusions of this manuscript will be made available by the authors, without undue reservation, to any qualified researcher.

## Author Contributions

HG was primary investigator of this study, overseeing all aspects of study design, data compilation, review of transcripts for accuracy, statistical analysis, and was the primary author for the entire manuscript. JC collaborated with HG to design the Spanish Nutricity tools, collect and analyse interviews, contributing to Table 2. AC was responsible for cultural accommodation of Spanish Nutricity tools as well as data collection and translation of data obtained from Spanish speakers. SP, JZ, YC, SH, BG, and DS contributed to study design, analysis, data interpretation, and discussion, contributing to all sections of the manuscript. All authors read and approved the final manuscript.

### Conflict of Interest Statement

The authors declare that the research was conducted in the absence of any commercial or financial relationships that could be construed as a potential conflict of interest.
