# Supplementary material for: Preliminary Investigation of a Mobile Nutrition Literacy Website for Parents and Young Children
Source: Front Nutr. 2018 Dec 18;5:129. doi: 10.3389/fnut.2018.00129 (PMC6305458; doi:10.3389/fnut.2018.00129)
Supplement: Supplementary file 1 [file Data_Sheet_1.docx]

Supplementary Material

Mixed Methods Formative Evaluation of a Mobile Nutrition Literacy Training Program for Parents and Young Children

**Heather D. Gibbs^1*^, Juliana Camargo^1^, Susana Patton^2^, Jamie Zoellner^3^, Yvonnes Chen^4^, Ana Paula Cupertino^5^, Susan Harvey^6^, Byron Gajewski^8^, Debra K. Sullivan^1^**

^1^Department of Dietetics & Nutrition, Mail Stop 4013, University of Kansas Medical Center, Kansas City, KS, 66160, United States

^2^Department of Pediatrics, University of Kansas Medical Center, Kansas City, KS, United States

^3^ Department of Public Health Science, University of Virginia, Charlottesville, VA, United States

^4^ School of Journalism and Mass Communications, University of Kansas, Lawrence, KS, United States

^5^John Theurer Cancer Center, Hackensack Meridian Health, Hackensack, NJ, United States

^6^Department of Health, Sport, and Exercise Sciences, University of Kansas, Lawrence, KS, United States

^7^ Department of Biostatistics, University of Kansas Medical Center, Kansas City, KS, United States

*** Correspondence:**Heather D. Gibbs
[hgibbs@kumc.edu](mailto:hgibbs@kumc.edu)

Table 1. Organization and Content of Nutricity, a Novel Mobile Nutrition Literacy Intervention

| Environment | Relevant Nutrition Literacy Constructs | Instructional Concepts Important to Construct | Educational Content | Practice/Skill Reinforcement |
| --- | --- | --- | --- | --- |
| Grocery Store | Food Label Reading  Consumer Skills | Finding nutrient information on food labels; Interpreting healthfulness of food labels; How to use ingredients lists | “Food Fits! Grocery Store Tour“ Video  Food Label Video (FDA) | Parents:Check Your Skills Quiz (choose between similar food items using product information, feedback provided)  Children: “Good Groceries“ game |
| Home | Nutrition & Health  Food Groups  Household Food Measurement  Feeding Behaviors | Understanding dietary guidelines and nutrition terms; Identifying food group categories; Building a healthy plate; Recommended portion sizes; “Division of Responsibility“ of parent/child eating roles | “Food Fits! Food Groups“ video  “Feeding Children Well“ Video | Parents: Check Your Skills Quiz (terms, food groups, division of responsibility; feedback provided)  Children: Interactive MyPlate game |
| Restaurant | Nutrition & Health  Food Groups  Household Food Measurement | Building a healthy plate at restaurants; Recommended portion sizes; Understanding menus | "Food Fits! Healthy Eating When Dining Out“ Video | Parents: Check Your Skills Quiz (menu selection)  Children: Find the Fruits & Veggies (Digital Seek & Find) |

Table 2. Content analysis of interviews with parent participants regarding thoughts on the Nutricity website.

| **Interview Question** | **Themes Uncovered by Interviews with Spanish Speakers (n=3)** | **Themes Uncovered by Interviews with English Speakers (n=3)** | **Potential Action/Implication** |
| --- | --- | --- | --- |
| Please tell us a little more on how useful you found the information to be in the Nutricity Website. | - Provided ideas for healthier food options - Taught how to build a meal with different food groups - Navigate grocery store shopping | - Provided ideas for healthier food options - Taught about food labels and food portions - Navigate grocery stores and restaurant menus | - Create user guide for website navigation - Focus more on food purchase and nutrition (grocery store and restaurant) |
| How engaging was the website? | - All content was engaging - Grocery store and food labels were most engaging - Games need to improve on children’s engagement | - All content was engaging - Food labels, food portions, parenting/eating skills were most engaging | - Improve on children’s game animation |
| How easy was the information to understand? | - Very easy to understand | - Very easy to understand | - No changes needed |
| Describe what you found to be the greatest benefit or advantage of using the Nutricity Website. | - Help to make healthier food choices - Access to nutrition information for children - Education on food labels | - Able to learn a lot in a short period of time. - Learn what and how to offer food to my child. - Increase awareness of choosing food and not choose only based on taste and craving. | - Fast and reliable source of nutrition education for parents and children |
| What did you find to be one of the disadvantages of using the Nutricity Website? | - None - Technical problem with one video | - None | - Improve accessibility on videos. |
| How would you rate the ease of navigating the website? (rating of 10 = very easy) Explain any problems you had while using the website. | - Rated 9.5 of 10 - Support to know where to start in the website - Improve format for children (options to select content beside drop down menus) | - Rated 10 of 10 - Understandable level for most children. | - Create user guide for website navigation - Improve interaction for young children. |
| How would you rate the quality of information in the website? (10=highest quality rating) | - Rated 9.3 of 10 | - Rated 10 of 10 | - NA |
| Describe how you used the website with your child. | - More interaction in the games. - Gave resource to teach children about nutrition and food. | - Parent select the topic and invite children to watch together - Child did not watch for long time (too young). | - Improve interaction on games for children. - Guide parents to play the games with children. |
| How would you rate your child’s level of engagement with the website? | - One rated 7.0 of 10 - Two did not have children present | - Little engaging - Engagement at home and older children may be different. | - Explore children’s abilities to play games alone vs. assisted by caregiver. |
| If you had access to the website, would you continue to use it? Why or why not? | - All would continue to use the website - Children learning about nutrition in an interactive way would spark curiosity to try new and healthy foods. | - All would continue to use the website. - Information about which foods and portion sizes to offer children. - Information for how to select food based on the food label. - Serve as a source of nutrition information in a way that is easy to access and understand. | - Source of nutrition education for children - Increase children’s curiosity of nutrition - Education on food quality, portion size, food purchase - Easy to access and understand. |
| What changes, if any, would you suggest to the website to make it more useful and engaging? | - More animation in the games - Images need to be bigger and bolder. - Have access through phone. | - No further changes. - Video to share information is preferred method. | - Improve game animation. - Improve image shape and format. - Provide access through phone - Provide more videos. |

**
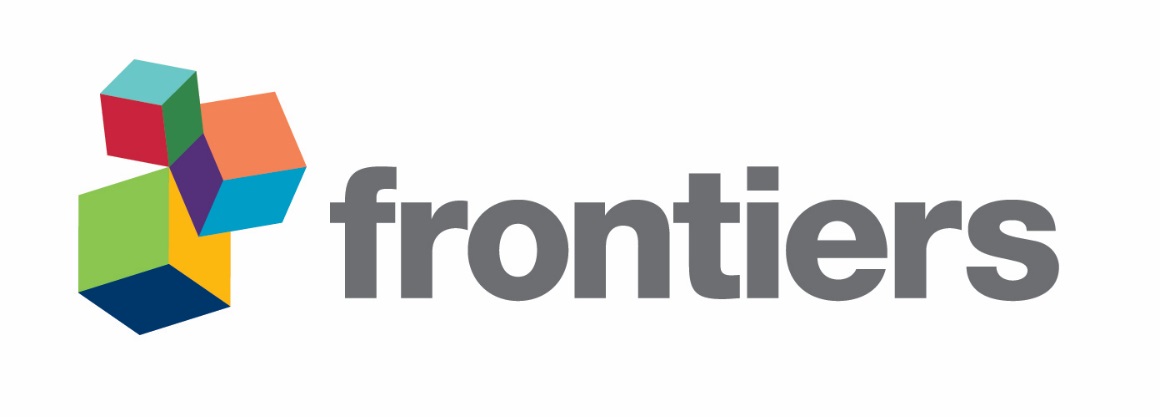
**
